# Early Diagnosis of ATTR-CM by Age- and Carpal Tunnel Biopsy-Guided Screening

**DOI:** 10.1016/j.jacadv.2026.102725

**Published:** 2026-04-09

**Authors:** Sie Kronborg Fensman, Bertil Thyrsted Ladefoged, Charlotte Hartig-Andreasen, Christina Stilling, Ali Hussein Jaber Mejren, Hans Christian Beck, Tor Skibsted Clemmensen, Steen Hvitfeldt Poulsen

**Affiliations:** a Department of Cardiology, Aarhus University Hospital, Aarhus, Denmark; b Institute of Health, Aarhus University, Aarhus, Denmark; c Department of Orthopedic Surgery, Aarhus University Hospital, Aarhus, Denmark; d Department of Pathology, Aarhus University Hospital, Aarhus, Denmark; e Odense Amyloidosis Center, Department of Clinical Biochemistry, Odense University Hospital, Odense, Denmark

**Keywords:** ATTR-CM, carpal tunnel syndrome, early diagnosis, screening

## Abstract

**Background:**

Transthyretin amyloid cardiomyopathy (ATTR-CM) is frequently preceded by carpal tunnel syndrome (CTS). The value of age- and biopsy-guided screening during CTS surgery for early detection of ATTR-CM remains uncertain.

**Objectives:**

We aimed to determine the proportion of amyloid deposition in tenosynovial biopsies, the proportion of ATTR-CM among biopsy-positive patients, and to compare disease severity between screening-detected and clinically diagnosed ATTR-CM patients.

**Methods:**

This prospective, multicenter cohort study enrolled men ≥65 and women ≥75 years undergoing surgery for idiopathic CTS and analyzed tenosynovial biopsies for amyloid deposition. Patients with amyloid-positive biopsies underwent cardiac workup, including echocardiography, bone scintigraphy, biomarker testing, and genetic testing to confirm ATTR-CM. A control cohort of contemporary clinically diagnosed wildtype ATTR-CM patients was included for comparison.

**Results:**

In total, 109 CTS patients were included (median age 79 years [IQR: 75-82]). Tenosynovial amyloid deposition was detected in 61/109 patients (56.0%; 95% CI: 46.1-65.5), of whom 52/61 patients completed cardiac evaluation. ATTR-CM was diagnosed in 9/52 patients (17.3%; 95% CI: 8.2-30.3; median age 83 years [IQR: 80-84]; male-to-female ratio 3.5:1; n = 7/2). Patients identified through screening had milder disease, 8/9 (89%) were in the National Amyloidosis Centre stage I compared to 26/47 (55%) among clinically diagnosed patients.

**Conclusions:**

Systematic age- and biopsy-guided screening during CTS surgery identified a high proportion of carpal amyloid deposition. Among amyloid-positive CTS patients, 1 in 6 was diagnosed with early-stage ATTR-CM. This approach demonstrated a higher yield in identifying ATTR-CM with mild disease burden and may serve as a tool for earlier diagnosis.

## Central Illustration

**Figure:**
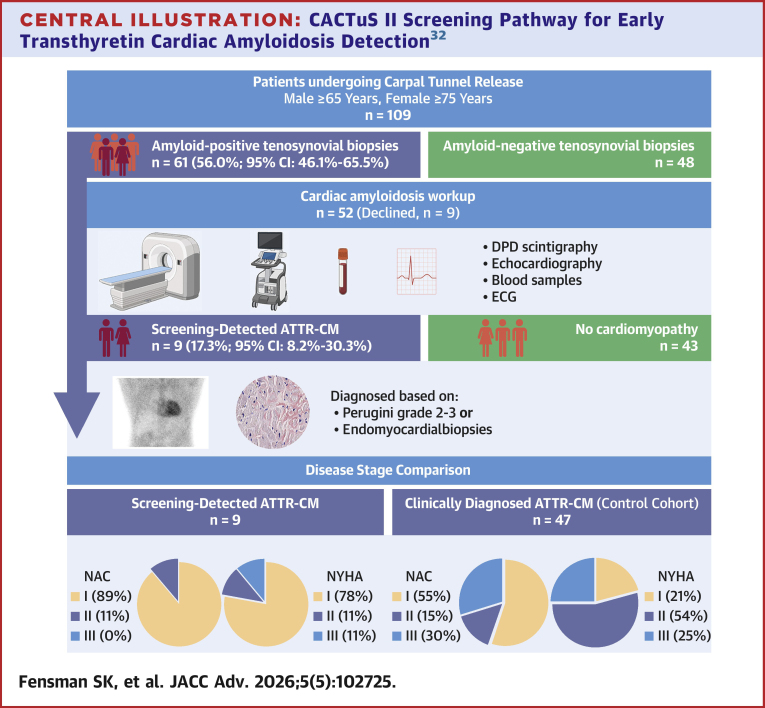

Transthyretin amyloid cardiomyopathy (ATTR-CM) is a restrictive cardiomyopathy characterized by progressive myocardial amyloid deposition, causing heart failure, arrhythmias, and reduced survival in elderly patients.^1^ Despite improved diagnostic work-up, ATTR-CM remains under-recognized, with diagnostic delays associated with worse disease severity and prognosis.^2^^,^^3^ Disease-modifying stabilizers are most effective in patients with less advanced disease, highlighting the importance of early diagnosis.^4^^,^^5^

Extracardiac manifestations often precede symptomatic ATTR-CM, with carpal tunnel syndrome (CTS) being the most common, affecting up to 80% of ATTR-CM patients.^6^^,^^7^ CTS typically precedes cardiac diagnosis by 5 to 10 years,^8^^,^^9^ making it a useful screening target for early detection of ATTR-CM.

Biopsies obtained during carpal tunnel release (CTR) surgery detect amyloid deposition in approximately 14% to 20% of CTS patients.^10^^,^^11^ However, prevalence increases with age and CTS severity.^12^ For CTS-guided screening of ATTR-CM, an age cutoff of 65 years has been proposed.^13^ The proportion of cardiac involvement at the time of CTR in an age-selected cohort remains unclear, and systematic screening strategies are lacking. The primary aim of this study was to determine the proportion of amyloid deposition in carpal tenosynovial biopsies and, among amyloid-positive patients, the proportion of ATTR-CM. The secondary aim was to compare screening-detected ATTR-CM patients with a cohort of contemporary clinically diagnosed wildtype (wt) ATTR-CM (ATTRwt-CM) patients with respect to clinical characteristics and disease stage.

## Methods

### Study design

We conducted a prospective multicenter cohort study including consecutive patients (men ≥65 years and women ≥75 years) undergoing surgery for idiopathic CTS at Aarhus University Hospital and 4 regional orthopedic centers in central Jutland between August 1, 2020, and May 30, 2025. Patients with prior amyloid disease, multiple myeloma, Waldenström macroglobulinemia, or monoclonal gammopathy of uncertain significance were excluded.

The study was conducted in accordance with the Declaration of Helsinki, approved by the Central Denmark Region Committees on Health Research Ethics (1-10-72-178-19), and registered at ClinicalTrials.gov (NCT04276220). Written informed consent was obtained from all participants.

### Screening algorithm

During open CTR, 2 to 3 tenosynovial tissue biopsies were collected, formalin-fixed, and stained with hematoxylin-eosin and Congo red. Amyloid deposits were confirmed by experienced pathologists using polarized light microscopy. All patients with amyloid-positive biopsies underwent cardiac workup within a median of 5 weeks [IQR: 4-6].

Amyloid typing by mass spectrometry of tenosynovial biopsies was performed in all ATTR-CM patients as well as on amyloid-positive biopsies from an age- and sex-matched set of participants.^14^

### Cardiac amyloid workup

Comorbidities and physical examination findings were recorded. A 12-lead electrocardiogram was performed. Blood samples included N-terminal pro-B-type natriuretic peptide (NT-proBNP), troponin I, creatinine, estimated glomerular filtration rate, free light chains, and monoclonal immunoglobulin by immunofixation. NT-proBNP <300 ng/L was considered normal. Troponin I was analyzed on a Siemens Atellica IM Analyzer, with institutional reference limits defined as <33 ng/L for women and <94 ng/L for men. Urine was tested for monoclonal immunoglobulin.

Transthoracic echocardiography was performed using the Vivid E95 or E9 (GE Healthcare) with a 3.5 MHz 4D transducer, and analyses were conducted in EchoPAC software. Standard measurements of cardiac structure and function were obtained in accordance with current guidelines.^15^ *99mtechnetium-labeled bone scintigraphy* was performed on all patients with amyloid-positive tenosynovial biopsies. Patients received 750 MBq of 99mtechnetium-labeled bone scintigraphy intravenously and were imaged 3 hours later using hybrid SPECT/CT systems (Siemens Symbia/Intevo/pro.Specta series or GE Discovery models) and cardiac SPECT/CT. Myocardial uptake was graded 0 to 3 using the Perugini criteria.^16^ ATTR-CM was diagnosed according to international nonbiopsy criteria.^17^ A Perugini grade of 2 or 3 was considered diagnostic for ATTR-CM; grade 1 required confirmatory endomyocardial biopsy; grade 0 excluded ATTR-CM. Disease severity was assessed using the National Amyloidosis Centre (NAC) staging system.^18^ Functional status and symptom burden were assessed using the NYHA functional class. ATTR-CM subtype was determined by genetic testing.

### Clinically diagnosed ATTRwt-CM control cohort

Newly clinically diagnosed ATTRwt-CM patients, who were able to undergo study-related assessments, served as controls. Enrollment was paced by the screening enrollment rate, aiming to include approximately 5 clinically diagnosed ATTRwt-CM patients for each screening-detected patient. Clinical controls were enrolled at Aarhus University Hospital between May 1, 2020, and June 30, 2024, using identical diagnostic criteria and cardiac workup as for the screening-detected patients.

### Statistics

Normality of data was assessed using the D’Agostino and Pearson test as well as visual inspection of histograms and Q-Q plots. Continuous variables are presented as medians (IQR). Categorical variables are presented as counts (%). CIs for proportions were calculated using the exact binomial (Clopper-Pearson) method. Table 1, Table 2, Table 3 are descriptive; therefore, broad hypothesis-free testing was intentionally avoided, and *P* values are not reported. Selected prespecified comparisons relevant to the study’s aims are tested using Mann-Whitney U test for continuous variables, and Pearson chi-square test, or Fisher exact test for categorical variables, as appropriate. Statistical analyses were performed using STATA 19 software (StataCorp LP). All analyses used 2-sided *P* values. A *P* value of <0.05 was considered statistically significant.

**Table 1:** Baseline Characteristics of the Total Study Cohort

	Total Study Population (N = 109)	Amyloid Positive Tenosynovial Biopsy (n = 61)	Amyloid Negative Tenosynovial Biopsy (n = 48)
Demographics			
Age, y	79 [65-93]	80 [65-93]	77 [66-90]
Male	74 (67.9)	42 (68.9)	32 (66.7)
BMI, kg/m^2^	27.1 (24.7-29.7)	27.3 (25.3-29.8)	26.8 (24.2-28.4)
Comorbidities			
Hypertension	72 (66.1)	41 (67.2)	31 (64.6)
Diabetes	16 (14.7)	8 (13.1)	8 (16.7)
Ischemic heart disease	12 (11.0)	6 (9.8)	6 (12.5)
Atrial fibrillation/flutter	11 (10.1)	6 (9.8)	5 (10.4)
Aortic stenosis	3 (2.8)	3 (4.9)	0 (0)
Pacemaker	2 (1.8)	1 (1.6)	1 (2.1)
Orthopedic comorbidities			
Bilateral CTS	61 (56.0)	38 (62.3)	23 (47.9)
Other orthopedic comorbidity^a^	50 (45.9)	36 (59.0)	14 (29.2)

BMI = body mass index; CTS = carpal tunnel syndrome.

Values are median [range], median (IQR: 25-75), or n (%).

a Patients suffering from trigger finger, spinal stenosis and/or tendon rupture.

**Table 2:** Characteristics of Patients With Amyloid-Positive Biopsies

	ATTR-CM (n = 9)	No cardiomyopathy (n = 43)
Demographics		
Age, y	83 [79-86]	79 [65-93]
Male	7 (77.8)	30 (69.8)
BMI, kg/m^2^	25.8 (24.8-28.0)	27.5 (25.7-30.8)
Comorbidities		
Hypertension	5 (55.6)	29 (67.4)
Diabetes	1 (11.1)	5 (11.6)
Ischemic heart disease	1 (11.1)	4 (9.3)
Atrial fibrillation/flutter	2 (22.2)	3 (7.0)
Aortic stenosis	2 (22.2)	1 (2.3)
Pacemaker	0 (0)	1 (2.3)
Orthopedic comorbidities		
Bilateral CTS	7 (77.8)	28 (65.1)
Other orthopedic comorbidity^a^	8 (88.9)	24 (55.8)
Biochemistry		
Creatinine, μmol/L	77 (76-105)	75 (63-88)
eGFR, mL/min/1.73 m^2^	78 (57-81)	80 (71-86)
Troponin I, ng/L	19 (11-27)	8 (5-11)
NT-proBNP, ng/L	576 (366-1454)	136 (52-307)
ECG		
ECG alterations^b^	3 (33.3)	2 (4.7)
Sokolow-Lyon value	19 (13-26)	19 (16-24)
Echocardiography		
IVS, mm	14 (13-15)	11 (10-12)
PW, mm	11 (10-11)	8 (7-9)
LV mass index, g/m^2^	110.9 (96.5-135.2)	78.5 (59.3-91.7)
LVEF, %	54 (37-54)	57 (54-61)
GLS, %	−14.4 (−16.2 to −8.9)	−17.2 (−19.7 to −16.0)
Apical Sparring Pattern	5 (62.5)	6 (16.2)
E/A ratio	0.73 (0.56-1.44)	0.74 (0.64-0.85)
E/e' ratio	10 (8-11)	9 (6-12)
LAVi, mL/m^2^	33 (26-34)	25 (18-32)
TAPSE, mm	21 (18-25)	23 (20-25)
TRG, mm Hg	23 (14-24)	27 (20-33)

ECG = electrocardiogram; eGFR = estimated glomerular filtration rate; GLS = global longitudinal strain; IVS = interventricular septum; LAVi = left atrial volume index; LV mass index = left ventricular mass index; LVEF = left ventricular ejection fraction; NT-proBNP = N-terminal pro-B-type natriuretic peptide; PW = posterior wall; TAPSE = tricuspid annular plane systolic excursion; TRG = tricuspid return gradient; other abbreviations as in Table 1.

Values are median [range], median (IQR: 25-75), or n (%).

a Patients suffering from trigger finger, spinal stenosis and/or tendon rupture.

b Low voltage, pseudo-infarction pattern or signs of left ventricular hypertrophy.

**Table 3:** Characteristics of Screening-Detected or Clinically Diagnosed ATTR-CM

	Screening-Detected ATTR-CM (n = 9)	Clinically Diagnosed ATTR-CM (n = 47)
Demographics		
Age, y	83 [79-86]	82 [60-92]
Male	7 (77.8)	45 (95.7)
BMI, kg/m^2^	25.8 (24.8-28.0)	25.3 (24.3-26.6)
NYHA functional class		
I	7 (77.8)	10 (21.3)
II	1 (11.1)	26 (55.3)
III	1 (11.1)	12 (25.5)
Biochemistry		
Creatinine, μmol/L	77 (76-105)	109 (85-147)
eGFR, mL/min/1.73 m^2^	78 (57-81)	56 (39-72)
Troponin I, ng/L	19 (11-27)	58 (38-78)
NT-proBNP, ng/L	576 (366-1454)	2,166 (1,294-4,282)
NAC class		
I	8 (88.9)	26 (55.3)
II	1 (11.1)	7 (14.9)
III	0 (0)	14 (29.8)
Echocardiography		
IVS, mm	14 (13-15)	17 (16-19)
PW, mm	11 (10-11)	13 (12-15)
LV mass index, g/m^2^	110.9 (96.5-135.2)	160.4 (128.6-180.3)
LVEF, %	54 (37-54)	48 (41-54)
GLS, %	−14.4 (−16.2 to −8.9)	−11.0 (−12.7 to −8.9)
Apical sparring pattern	5 (62.5)	41 (95.3)
E/A ratio	0.73 (0.56-1.44)	1.6 (1.0-2.6)
E/e' ratio	10 (8-11)	12.2 (9.6-16.7)
LAVi, mL/m^2^	33 (26-34)	44 (33-54)
TAPSE, mm	21 (18-25)	17 (14-19)
TRG, mm Hg	23 (14-24)	25 (22-33)

ATTR-CM = transthyretin amyloid cardiomyopathy; NAC = National Amyloidosis Centre; other abbreviations as in Tables 1 and 2.

Values are median [range], median (IQR: 25-75), or n (%).

## Results

### Proportion of amyloid-positive carpal biopsies and ATTR-CM

**Figure 1:**
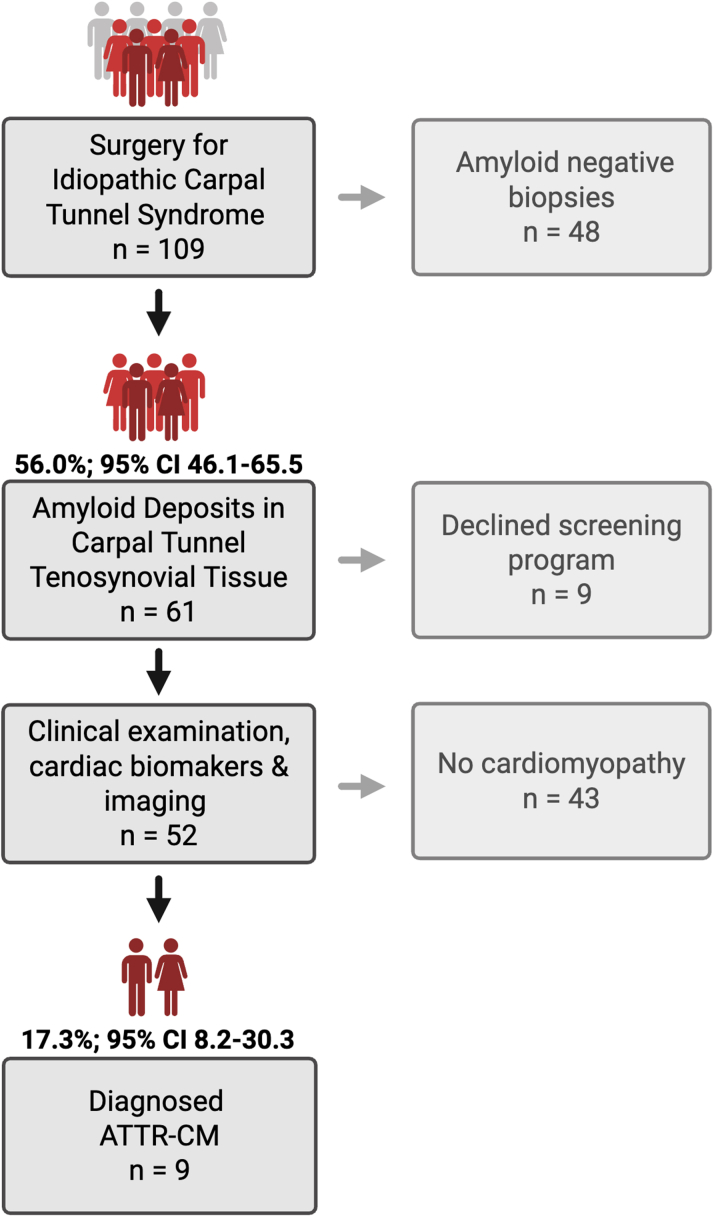
**Screening Pathway** Patient flow through the CACTuS II screening pathway. Patients aged ≥65 (men) or ≥75 years (women) undergoing carpal tunnel syndrome surgery were screened. Tenosynovial biopsies were stained with Congo red, and patients with confirmed amyloid underwent a standardized cardiac. The flow diagram highlights the proportion of amyloid deposits in tenosynovial tissue and those subsequently diagnosed with ATTR-CM. Created in Biorender.^32^ ATTR-CM = transthyretin amyloid cardiomyopathy.

**Figure 2:**
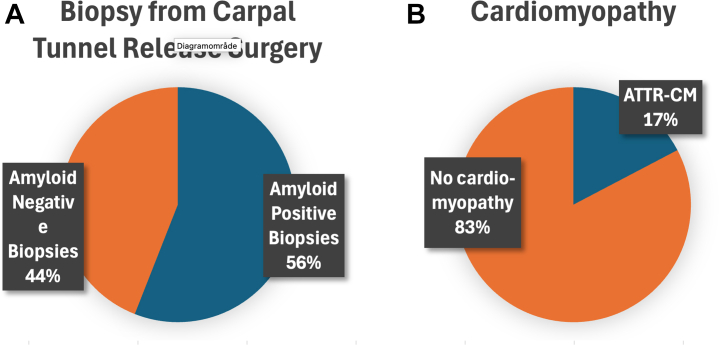
**Prevalence of Tenosynovial Amyloid and Transthyretin Cardiac Amyloidosis** Pie chart illustrating the distribution of (A) amyloid-positive and amyloid-negative tenosynovial biopsies and (B) ATTR-CM or no cardiomyopathy. Abbreviation as in Figure 1.

A total of 109 patients undergoing surgery for idiopathic CTS were included. The flow of participants is presented in Figure 1. Their median age at inclusion was 79 years (IQR: 75-82), and 74 of 109 (68%) were males. Amyloid deposition in the carpal tunnel tenosynovial tissue was confirmed in 61 of 109 patients (56.0%; 95% CI: 46.1%-65.5%). Nine of 61 patients (15%) with amyloid-positive carpal biopsies declined to proceed with the cardiac amyloidosis workup. There were no significant differences in baseline characteristics between patients who discontinued and those who completed the cardiac assessment. A total of 52 of 61 patients completed further cardiac workup, of whom 9 (17.3%; 95% CI: 8.2%-30.3%) had ATTR-CM. Their median age was 83 years (IQR: 80-84), and 7 of 9 (78%) were males. Among amyloid-positive male patients older than 75 years, cardiomyopathy was identified in 7 of 26 (26.9%) men. Figure 2 shows the distribution of biopsy results and the proportion of ATTR-CM among amyloid-positive patients.

Mass spectrometry confirmed transthyretin-type amyloid in all analyzed tenosynovial biopsies. This included all patients diagnosed with ATTR-CM (n = 9) and a comparison group of amyloid-positive patients without cardiac involvement (n = 12; 8 men, 4 women).

### Characteristics of patients with amyloid-positive tenosynovial biopsies

Table 1 summarizes patient characteristics stratified by amyloid status on tenosynovial biopsy. Patients with amyloid-positive biopsies were older (*P* = 0.001), whereas sex distribution was evenly balanced between the groups. Patients with amyloid-positive biopsies had more amyloid-related orthopedic comorbidities, such as trigger finger, spinal stenosis, and tendon rupture (*P* = 0.001). The prevalence of atrial fibrillation did not differ between the 2 groups.

### Characteristics of screening-detected ATTR-CM patients

Nine patients were diagnosed with ATTR-CM. Eight patients with Perugini grade 2 to 3 uptake and 1 patient with grade 1 uptake, in whom ATTRwt-CM was confirmed by endomyocardial biopsy, revealing transthyretin amyloid deposition.

Two screening patients had Perugini grade 1, but ATTR-CM could not be confirmed. Both patients had normal NT-pro-BNP and troponin I levels. One had early echocardiographic signs of ATTR-CM (left ventricular [LV] hypertrophy and mildly reduced global longitudinal strain) and underwent endomyocardial biopsy, which showed no amyloid deposition. The other was a female patient with no signs of cardiomyopathy, normal interventricular wall thickness (8 mm), normal systolic and diastolic function, and a normal electrocardiogram.

Genetic testing showed that 8 patients had ATTRwt-CM, and 1 female patient had hereditary ATTR-CM carrying the rare pathogenic mutation Ala65Ser.^19^

Of the 9 patients with ATTR-CM, 2 (22%) had NT-proBNP levels within the normal range, and 8 (89%) had troponin I levels within the normal range. Two (22%) patients reported mild to moderate dyspnea (NYHA functional class II and III), whereas the remaining 7 (78%) were asymptomatic. Eight patients had mild disease (NAC I), whereas 1 patient had moderate disease (NAC II). Under current national treatment recommendations, 4 (44%) would be considered eligible for disease-modifying therapy.

Table 2 summarizes the characteristics of patients with ATTR-CM and those without proven cardiomyopathy. Patients diagnosed with ATTR-CM were older (*P* = 0.020) than those without cardiomyopathy, whereas sex was equally distributed between the 2 groups. Atrial fibrillation, aortic stenosis, and orthopedic amyloid-related comorbidities tended to be more common among those with ATTR-CM. Troponin I and NT-proBNP levels were higher in patients with ATTR-CM than in those without cardiomyopathy (*P* = 0.001 and *P* = 0.004, respectively). Echocardiographic evaluation revealed structural cardiac changes in ATTR-CM patients, including increased interventricular septal wall thickness (*P* = 0.002), higher LV mass index (*P* = 0.001), and impaired systolic function, with reduced LV ejection fraction (*P* = 0.003) and LV global longitudinal strain (*P* = 0.007).

### Comparison of screening-detected versus clinically diagnosed ATTR-CM

**Figure 3:**
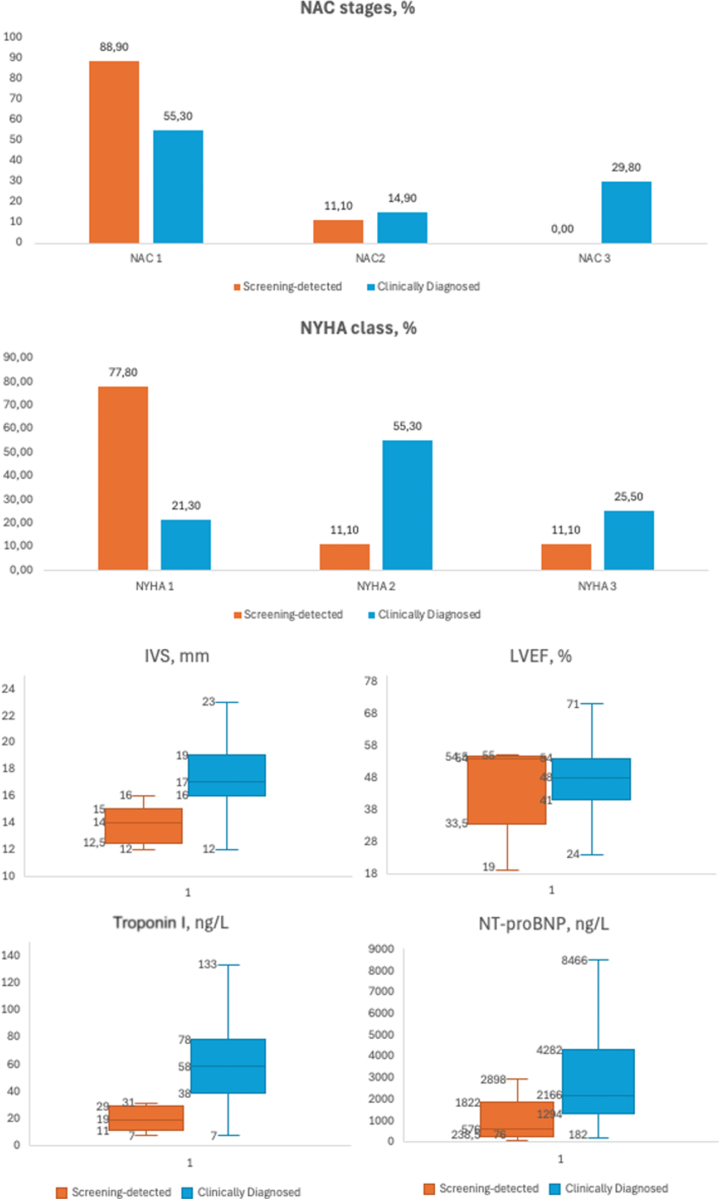
**Comparing Screening-Detected and Clinically Diagnosed Transthyretin Cardiac Amyloidosis** This figure compares disease stage, functional class, cardiac structure, and biomarker levels in screening-detected vs clinically diagnosed ATTR-CM patients. Exact binomial 95% confidence intervals for NAC stage and NYHA class are provided in Supplemental Table 2. Boxplots display the median (center line), interquartile range (25th-75th percentiles; box), and whiskers extending to the most extreme values within 1.5 times the interquartile range. Values beyond the whiskers represent outliers. Comparisons between groups showed IVS *P* < 0.001; LVEF *P* = 0.78; Troponin I *P* < 0.001; NT-proBNP *P* = 0.006. IVS = interventricular septum; LVEF = left ventricular ejection fraction; NAC = National Amyloidosis Centre; NT-proBNP = N-terminal pro-B-type natriuretic peptide.

From the outpatient clinic, 47 patients with a newly established ATTRwt-CM diagnosis were enrolled (Table 3, Figure 3), representing approximately one-third of the 131 newly diagnosed ATTRwt-CM patients at our institute during the study period. Screening-detected and clinically diagnosed ATTR-CM patients had similar ages. The screening-detected cohort had a higher proportion of women (2 of 9 [22%]) compared to the clinically diagnosed group (2 of 47 [4%]). Screening-detected patients were generally less symptomatic, with 7 of 9 (78%) in NYHA functional class I, compared to 10 of 47 (21%) in the clinically diagnosed cohort. Biomarkers also differed between groups: screening-detected patients had numerically higher estimated glomerular filtration rate (*P* = 0.062) and lower troponin I (*P* < 0.001) and NT-proBNP (*P* = 0.006) levels. Among screening-detected patients, 8 of 9 (89%) were classified as NAC stage I, 1 of 9 (11%) as NAC stage II, and none as NAC stage III. In contrast, among clinically diagnosed patients, 26 of 47 (55%) were in NAC stage I, 7 of 47 (15%) in NAC stage II, and 14 of 47 (30%) in NAC stage III.

Structural cardiac involvement was less advanced in screening-detected patients; they had milder LV hypertrophy (interventricular septal wall thickness, *P* < 0.001) and less impaired systolic and diastolic function compared to clinically diagnosed patients.

In a post hoc sensitivity analysis restricted to age-matched male patients, screening-detected ATTR-CM consistently showed milder disease across biomarkers, echocardiographic measures, and NAC stage (Supplemental Table 1).

### Follow-up

Patients with amyloid-positive tenosynovial biopsies who underwent cardiac evaluation but were not diagnosed with ATTR-CM were followed for a mean of 2.3 ± 1.4 years (range 0.7-5.4). Among these 43 patients, none were subsequently diagnosed with ATTR-CM, 2 (4.7%) died from noncardiac causes, and 8 (18.6%) had cardiac-related hospitalizations and/or cardiac outpatient assessments.

## Discussion

Systematic age- and biopsy-guided screening during surgery for idiopathic CTS in elderly patients identified a high proportion of tenosynovial amyloid deposition and enabled detection of predominantly early-stage ATTR-CM.

### Tenosynovial amyloid deposition and ATTR-CM

Our study demonstrates that amyloid deposition in the carpal tunnel is common among elderly patients undergoing CTR, 56% had amyloid-positive tenosynovial biopsies, and 17% of these patients had concurrent ATTR-CM after an immediate cardiac amyloidosis workup.

Sperry et al.^20^ provided a proof of concept for CTS-based screening, reporting amyloid deposition in 10% of carpal biopsies from patients undergoing CTR (median age 68 years [IQR: 61-74]). Since then, studies have aimed to refine screening strategies to increase predictive accuracy. In an elderly cohort, Maehara et al. found amyloid deposition in 56% of carpal biopsies, consistent with our results, whereas younger CTS populations show lower prevalences. In most prior studies, systematic cardiac evaluation was absent or incomplete, resulting in limited and inaccurate estimation of cardiac involvement.^10^, ^11^, ^12^, ^13^^,^^21^ Data suggest that the prevalence of amyloid-positive tenosynovial biopsies and coexisting ATTR-CM is influenced by age and sex. Amyloid-positive biopsies represent a strong clinical marker of potential ATTR-CM in elderly CTS patients.

This study identified 1 patient with hereditary ATTR-CM, highlighting that systematic screening may also uncover unrecognized hereditary disease. Previous screening studies identified only ATTRwt patients. If this observation is confirmed in future screening cohorts, it could expand detection of hereditary cases and facilitate identification of additional genetic carriers.

### Performance of biomarkers in CTR-based ATTR screening

In the present study, a substantial proportion of screening-detected ATTR-CM patients had normal cardiac biomarkers at diagnosis. The CACTuS I study likewise observed biomarker-negative cases among screening-detected patients.^22^ Noory et al.^23^ identified screening-detected patients with higher troponin T and NT-proBNP levels and introduced biomarker cutoffs as reliable negative predictors. However, their cohort was evaluated 5 to 15 years after bilateral CTR, whereas patients in the present study were assessed immediately postoperatively. Takashio et al.^21^ provided an example of a biomarker (troponin T) and LV hypertrophy-guided screening for ATTR-CM in patients with amyloid-positive carpal biopsies. This approach led to scintigraphy in 10% of the screened population, of whom half were positive for ATTR-CM. Thus, a biomarker-based approach might be effective in identifying more advanced cases. However, it may miss a substantial proportion of the earliest and preclinical cases, as it restricts patient selection for further cardiac evaluation. This limits the value of biomarker-based screening strategies. In settings with limited access to bone scintigraphy, a stepwise, multiparametric diagnostic approach incorporating echocardiography, electrocardiogram, and biomarkers may serve as an initial triage strategy before referral for confirmatory scintigraphy.^24^

### Sex distribution in ATTRwt-CM

Beyond early disease detection, screening may also broaden the recognized clinical phenotype of ATTRwt-CM, particularly regarding the distribution of female patients. In our cohort, a higher proportion of women were identified through screening than through usual clinical pathways. This pattern is consistent with findings from Chan et al,^25^ who reported that systematic screening with nuclear imaging increased the proportion of women diagnosed with ATTRwt-CM to 27.8%. Historically, women have been estimated to represent only 10% of cases,^26^ although these studies are small, this suggests that conventional pathways may overlook female patients. Supported by a meta-analysis by Kroi et al,^26^ which found that autopsy-based studies, unaffected by referral bias, reported a female proportion of 30.7%. Although postmortem evaluation does not directly translate to clinical cohorts, this highlights the persistent diagnostic challenge of ATTRwt-CM in women. Recent studies also indicate that the proportion of female ATTRwt-CM patients increases with age.^26^^,^^27^ Therefore, the use of age- and sex-specific thresholds appears to improve detection while reducing unnecessary evaluations.

### Implications of screening

ATTR-CM fulfills the key screening principles outlined by the World Health Organization.^28^ It is an important health problem that affects patients’ quality of life and survival. Reliable, low-burden confirmatory diagnostic testing is available, along with disease-modifying therapies that improve outcomes. The socioeconomic implications of screening remain uncertain. Amyloid detection from CTR surgery can be performed safely and at low cost, supporting their role as an entry point for targeted cardiac screening in high-risk populations.

Disease-modifying therapies for ATTR-CM are associated with substantial costs, and early identification of patients may increase the number of patients considered for long-term treatment.^4^^,^^5^ Trails have reported numbers needed to treat of approximately 5 to 7 to prevent heart failure hospitalization or death,^29^ and future changes in drug pricing, including loss of patent protection and increased competition, may influence the cost-benefit balance over time. Optimal screening strategies should consider the potential psychological impact of screening. As with all screening strategies, earlier detection must be interpreted cautiously, considering potential lead-time bias, in which patients identified at an earlier disease stage may appear to have better outcomes despite unchanged prognosis. The present study was not designed to assess prognosis, and earlier detection should not be interpreted as evidence of improved survival.

### Identification of early ATTR-CM

Early ATTR-CM detection enables timely guideline-based treatment initiation. Identification of preclinical ATTR-CM may provide insights into progression rates toward overt cardiomyopathy and help define populations for future preventive trials, similar to ACT-EARLY, to evaluate whether earlier intervention can delay or prevent symptomatic disease.^30^

Appropriate re-evaluation intervals and indications remain uncertain. During a mean follow-up of 2.3 ± 1.4 years, no patients with amyloid-positive tenosynovial biopsies without cardiomyopathy developed ATTR-CM. Repeat cardiac assessment after approximately 5 years, or earlier if cardiac symptoms are present, may be reasonable; however, no evidence-based recommendations currently exist, and prospective data are needed.

### Study Limitations

Most importantly, no screening log was kept; thus, selection bias must be considered when interpreting the reported prevalence estimates. We are unable to estimate the total number of CTS surgeries performed during the study period because patients were enrolled across multiple centers with varying inclusion periods. Different age cutoffs between sexes may contribute to a remaining under-representation of women. The absence of long-term follow-up prevents assessment of how many patients with amyloid-positive tenosynovial biopsies will progress to cardiac amyloidosis. The increasing use of endoscopic CTR might affect the future feasibility of biopsy-based screening. However, minor modifications to standard endoscopic techniques permit tenosynovial sampling, and have enabled the detection of tenosynovial amyloid deposition in biopsies from endoscopic CTR.^31^ This approach requires further evaluation. All participants were Caucasian, limiting generalizability.

## Conclusions

In an age- and sex-selected CTS cohort, over half (56.0%) had amyloid-positive tenosynovial biopsies. Among these, 1 in 6 (17.3%) were diagnosed with ATTR-CM (Central Illustration). Systematic biopsy-guided screening identified disease substantially earlier than routine clinical practice, enabling close monitoring of preclinical cases and timely initiation of disease-modifying therapies. This early detection framework also defines a population suited for future studies of preclinical disease modification.

Perspectives **COMPETENCY IN MEDICAL KNOWLEDGE:** Systematic age- and biopsy-guided screening during CTR within an existing surgical pathway can identify ATTR-CM at an earlier disease stage than routine clinical pathways. Early identification allows close disease monitoring and timely initiation of disease-modifying therapy in accordance with current guidelines.

**TRANSLATIONAL OUTLOOK:** Early detection provides an opportunity to study progression from preclinical ATTR-CM to overt cardiomyopathy and to evaluate whether earlier therapeutic intervention can delay or prevent disease onset.

**Central Illustration:**
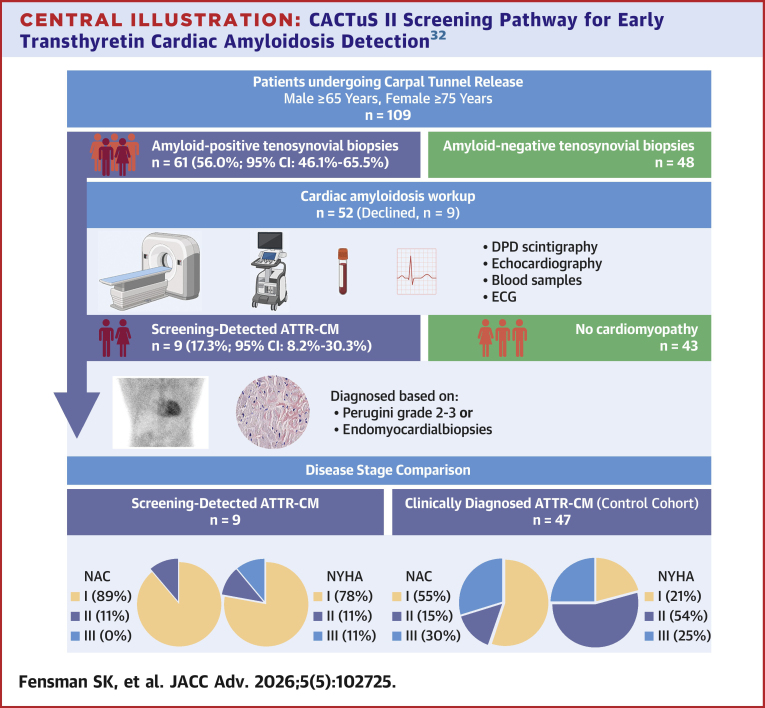
**CACTuS II Screening Pathway for Early Transthyretin Cardiac Amyloidosis Detection**^32^ Systematic biopsy-guided screening during carpal tunnel syndrome (CTS) surgery in patients aged ≥65 (men) or ≥75 years (women) identified a high prevalence of tenosynovial amyloid. Among patients with amyloid-positive biopsies, cardiac workup revealed a substantial proportion with early-stage ATTR-CM. This demonstrates how CTS surgery provides an accessible, low-cost opportunity to identify early-stage ATTR-CM and facilitate timely initiation of disease-modifying therapy before functional decline occurs. ATTR-CM = transthyretin amyloid cardiomyopathy; DPD = 99mtechnetium-labeled bone scintigraphy; ECG = electrocardiogram; NAC = National Amyloidosis Centre.

## Funding support and author disclosures

This study was supported by research funding from Novo Nordisk A/S. Dr Fensman has received research support from Novo Nordisk A/S; she received educational fee and travel support for conference participation from 10.13039/100004319Pfizer; and she received speaker honorarium from 10.13039/100004325AstraZeneca. Dr Ladefoged has received speaker fee from 10.13039/100004319Pfizer and 10.13039/100004326Bayer. Dr Jaber Mejren has received travel support for conference participation from 10.13039/100004319Pfizer. Dr Poulsen has received research support from Novo Nordisk A/S; and received consulting fees from 10.13039/100004319Pfizer, 10.13039/100004325AstraZeneca, 10.13039/100006400Alnylam, 10.13039/100014941Cytokinetics, BridgeBio, and Novo Nordisk A/S. All other authors have reported that they have no relationships relevant to the contents of this paper to disclose.
