# Supplementary material for: Early Diagnosis of ATTR-CM by Age- and Carpal Tunnel Biopsy-Guided Screening
Source: JACC Adv. 2026 Apr 9;5(5):102725. doi: 10.1016/j.jacadv.2026.102725 (PMC13091353; doi:10.1016/j.jacadv.2026.102725)
Supplement: Supplemental Material [file mmc1.docx]

**Supplement**

**Matched Control Cohort**

To address potential confounding by age and sex in the comparison between screening-detected and clinically diagnosed ATTRwt-CM, we performed a sensitivity analysis restricted to male patients with comparable age distributions. This approach was chosen because women were few in the clinically diagnosed cohort and exhibited a wider age span, precluding meaningful matching across sex strata.

Male patients from the screening-detected cohort were age-matched to clinically diagnosed male patients using a 1:3 ratio, yielding 7 screening-detected and 21 clinically diagnosed patients with similar median ages. No additional matching on disease severity, biomarkers, or comorbidities was performed.

Clinical characteristics, biomarkers, echocardiographic parameters, and NAC stage distributions in the age-matched male cohorts are presented in Supplementary Table S1. Screening-detected patients remained less symptomatic, with a higher proportion in NYHA class I than clinically diagnosed patients. All screening-detected patients in the age-matched male cohort were classified as NAC stage I, whereas clinically diagnosed patients had a broader distribution across NAC stages. Troponin I (p = 0.007) and NT-proBNP (p = 0.047) remained lower, and eGFR (p = 0.075) continued to trend higher. Structural cardiac involvement remained less advanced, with significantly lower interventricular septal wall thickness (p = 0.004).

Overall, the findings of this sensitivity analysis were consistent with the main analysis, supporting that earlier disease stage among screening-detected ATTRwt-CM patients is not solely explained by differences in age or sex, but reflects earlier disease identification through systematic screening.

Supplemental Table S1:

**Characteristics of cardic amyloidosis in age-matched males**

ATTR-CM, transthyretin amyloid cardiomyopathy; BMI, body mass index; eGFR, estimated glomerular filtration rate; GLS, global longitudinal strain; IVS, interventricular septum; LAVi, left atrial volume index; LV mass index, left ventricular mass index; LVEF, left ventricular ejection fraction; NAC, National Amyloidosis Centre; NT-proBNP, N-terminal pro-B-type natriuretic peptide; NYHA, New York Heart Association; PW, posterior wall; TAPSE, tricuspid annular plane systolic excursion; TRG, tricuspid return gradient.

Age is presented as median [range]; numerical variables as median (IQR 25–75); and categorical variables as counts (%).

^a^ Missing data from one patient

Supplemental Table S2:

**NYHA and NAC stage distribution with exact binomial 95% confidence intervals**

Confidence intervals were calculated using the exact binomial (Clopper–Pearson) method.

NAC, National Amyloidosis Centre; NYHA, New York Heart Association

| **Supplementary Table 1** | | |
| --- | --- | --- |
|  | Screening-detected Cardiac Amyloidosis | Clinically Diagnosed Cardiac Amyloidosis |
|  | n = 7 | n = 21 |
| Demographics |  |  |
| Age, years | 83 [79-86] | 83 [77-90] |
| BMI, kg/m^2^ | 27.2 (25.3-28.1) | 25.3 (24.3-26.1) |
| NYHA class |  |  |
| NYHA class I | 7 (85.7) | 5 (23.8) |
| NYHA class II | 0 (0) | 10 (47.6) |
| NYHA class III | 1 (14.3) | 6 (28.6) |
| Biochemistry |  |  |
| Creatinine, μmol/L | 77 (76-105) | 100 (85-147) |
| eGFR, mL/min/1.73 m^2^ | 78 (57-81) | 62 (39-72) |
| Troponin I, ng/L | 19 (11-31) | 55 (46-101) |
| NT-proBNP, ng/L | 576 (366-1454) | 2246 (1414-4802) |
| NAC class |  |  |
| NAC class I | 7 (100) | 12 (57.1) |
| NAC class II | 0 (0) | 3 (14.3) |
| NAC class III | 0 (0) | 6 (28.6) |
| Echocardiography |  |  |
| IVS, mm | 14 (13-15) | 17 (16-19) |
| PW, mm | 11 (10-12) | 14 (12-15) |
| LV mass index, g/m^2^ | 131.4 (96.5-135.2) | 148.1 (130.3-172.3) |
| LVEF, % | 54 (30-55) | 48 (44-55) |
| GLS, % | -13.0 (-17.1- -8.0) | -10.4 (-12.1- -7.7) |
| Apical Sparring Pattern, n (%) | 5 (71.4) | 20 (100%) ^a^ |
| E/A ratio | 0.92 (0.71-1.78) | 1.7 (1.2-2.8) |
| E/e' ratio | 9 (8-14) | 14 (7-19) |
| LAVi, mL/m^2^ | 34 (15-36) | 47 (37-55) |
| TAPSE, mm | 21 (17-25) | 17 (15-19) |
| TRG, mmHg | 23 (14-24) | 23 (21-31) |

| **Supplementary Table 2** | | | | | | |
| --- | --- | --- | --- | --- | --- | --- |
| Variable | Category | n | N | Percent (%) | 95% CI Lower | 95% CI Upper |
| Screening-detected ATTR-CM | | | | | | |
| NYHA | I | 7 | 9 | 77,8 | 40,0 | 97,2 |
| NYHA | II | 1 | 9 | 11,1 | 0,3 | 48,3 |
| NYHA | III | 1 | 9 | 11,1 | 0,3 | 48,3 |
| NAC | I | 8 | 9 | 88,9 | 68,4 | 99,7 |
| NAC | II | 1 | 9 | 11,1 | 0,3 | 48,3 |
| NAC | III | 0 | 9 | 0,0 | 0,0 | 33,6 |
| Clinically diagnosed ATTR-CM | | | | | | |
| NYHA | I | 10 | 47 | 21,3 | 10,7 | 35,7 |
| NYHA | II | 26 | 47 | 55,3 | 40,1 | 69,8 |
| NYHA | III | 12 | 47 | 25,5 | 13,9 | 40,4 |
| NAC | I | 26 | 47 | 55,3 | 40,1 | 69,8 |
| NAC | II | 7 | 47 | 14,9 | 6,2 | 28,3 |
| NAC | III | 14 | 47 | 29,8 | 17,3 | 44,9 |
